# Spatial Distribution, Source Identification, and Potential Ecological Risk Assessment of Heavy Metal in Surface Sediments from River-Reservoir System in the Feiyun River Basin, China

**DOI:** 10.3390/ijerph192214944

**Published:** 2022-11-13

**Authors:** Shengnan Zhu, Zengchuan Dong, Bohua Yang, Guangen Zeng, Yupeng Liu, Yuejiao Zhou, Jinyu Meng, Shujun Wu, Yiqing Shao, Junfei Yang, Xiao Guo

**Affiliations:** 1 College of Hydrology and Water Resources, Hohai University, Nanjing 210098, China; 2 Wenzhou Hydrology Management Center, Wenzhou 325000, China; 3 State Key Laboratory of Hydrology and Water Resources and Hydraulic Engineering, Nanjing Hydraulic Research Institute, Nanjing 210029, China; 4 Changzhou Tianning District Agriculture and Rural Bureau, Changzhou 213000, China

**Keywords:** heavy metals, surface sediments, potential ecological risk, Feiyun River Basin, river–reservoir system

## Abstract

To investigate the pollution characteristics of the surface sediments of the river–reservoir system in the Feiyun River basin, a sediment heavy metal survey was conducted for the first time in the Feiyun River basin. Surface sediments from 21 sampling sites in the Feiyun River basin were collected, and the concentrations and spatial distribution characteristics of 15 heavy metals (Cr, Ni, Cu, Zn, As, Cd, Pb, Mn, V, Co, Mo, Sb, W, Fe, and Se) were analyzed. Three heavy metal ecological risk assessment methods were used to evaluate the potential risks of heavy metals in sediments, and the sources of major heavy metals were traced by correlation analysis and principal component analysis. The results show that (1) the average concentration of heavy metals (As) (212.64 mg/kg) and (Sb) (4.89 mg/kg) in Feiyun River Basin is 33.3 and 6.89 times the background value of Zhejiang Province; the overall spatial distribution of heavy metals is: the mainstream of Feiyun River > Zhaoshandu Reservoir > Shanxi Reservoir, thereby, the pollution is relatively significant; (2) by processing the geo-accumulation index and enrichment index methods, As and Sb are classified as ‘severely polluted’, ‘moderately severely polluted’ and ‘severely polluted’, ‘very severe polluted’ respectively; (3) the potential ecological index evaluates the surface sediments in the Feiyun River Basin as a very high risk level, the main environmental risk factors are As, Sb, Cd and Mo; (4) the principal component analysis results show that the heavy metals in the sediments of the Feiyun River Basin may be mainly affected by human activities such as sewage from domestic and agricultural activities, mining and smelting, and the others are affected by natural factors.

## 1. Introduction

With the rapid development of industrialization and socio-economic, heavy metal pollution in aquatic ecosystems has become a global problem, in which heavy metal pollution in river systems is of great concern due to its inherent toxicity, numerous sources, bioaccumulation, and persistence in the environment [1,2]. As a major “reservoir” and important carrier in the aquatic environment, more than 85% of heavy metals are eventually deposited and enriched in the surface sediments [3,4]. Natural conditions (weathering of rocks, wet and dry deposition, rainfall-runoff) and human activities (production and domestic sewage, industrial and agricultural sewage, and mining) can lead to the migration and deposition of heavy metals in the river system. When the concentration of heavy metals reaches a threshold, it can lead to malnutrition and even death of aquatic organisms, and also seriously threaten the health of animals and humans [5,6]. Heavy metals are important pollutants in the water environment and the pollution status of a water body can be measured by the concentration status of the pollutants, so understanding the heavy metals in the surface sediments of river systems is essential for water environmental safety assessment. In recent years, studies have been conducted to understand the sources, transport, and accumulation of heavy metals in river sediments [7,8]. Sediments can be used as indicators of heavy metal contamination in the aquatic environment and can provide useful tools for understanding the geochemical interactions, source characteristics, and potential risk status of heavy metals in sediments. Moreover, many researchers have studied the status of heavy metal pollution in watershed sediments, focusing mainly on investigating the spatial distribution of heavy metals in sediments, assessing their potential ecological risks, and identifying their possible sources [9,10,11,12,13,14,15]. The environmental impact of heavy metals in surface sediments of water bodies has been studied mainly in large lakes and rivers, but little research has been reported on the characteristics of surface sediment heavy metal concentrations, potential ecological risks, and sources in river-reservoir system type basins.

Just like river and lake systems, river reservoir systems are where rivers are connected to reservoirs [16,17]. These systems play an important role in the transformation and transport of land-derived materials, the deposition of suspended materials, and chemical changes [18,19]. This system evolved from a primitive river type to a dual-use river and reservoir type after the construction of dams, leading to increased sedimentation in the waters and land areas, and the sedimentary soils in the core area became a gathering place for heavy metals, etc. in the upper reaches of the watershed and surrounding areas. Especially after the water level was raised, the water area of the core area of the reservoir in the system expanded, resulting in the increased environmental risk of heavy metals in water bodies, sediments, substrates, and surrounding soils in the water source. Furthermore, the dam built for the reservoir alters the natural state of the river, greatly changes the environmental conditions of the water area, and affects the biogeochemical process of the river [20]. These changes affect the distribution and transport of heavy metals in river sediments. The purpose of heavy metal assessment is to determine the sources of heavy metals [21]. Monitoring studies on the enrichment of heavy metals in sediments in the interaction zone of river-reservoir systems can help to explain the origin and nature of heavy metals in rivers and reservoirs [22,23]. Therefore, it is necessary to study the levels, distribution, sources, and ecological risks of heavy metals in sediments of river-reservoir systems.

Feiyun River is an important river located in the southeast of Zhejiang Province. The Feiyun River Basin covers a variety of water types such as mountainous rivers, large-sized reservoirs, plain rivers, tidal sections, etc. The Shanxi-Zhaoshandu Reservoir in the basin is a national drinking water source, where Shanxi Water Conservancy Hub Project is located. The river–reservoir system is also in the middle reaches of the Feiyun River basin. Feiyun River plays an important role in the safety of drinking water, economic and social development, and sustainable development of the ecological environment [24]. However, high urbanization and rapid economic development have made it increasingly polluted, with a large number of pollutants entering the river–reservoir systems. Thus, the evaluation of heavy metals in the sediments of these river–reservoir systems is beneficial to determine the impact of pollution on them. At present, it is mainly to study the assessment of heavy metals at the mouth of the Feiyun River basin, but not to study the river–reservoir system of the Feiyun River basin [25,26]. Therefore, this study is the first evaluation of heavy metal pollution in the surface sediments of the riverbank system in the Feiyun River basin, which is important for understanding the level, spatial distribution, source identification, and potential ecological risk analysis of heavy metals in the river–reservoir system in the Feiyun River basin.

The main objectives of this study are (1) to analyze the level and spatial distribution of heavy metals in the sediments of river–reservoir systems, (2) to evaluate the pollution level and enrichment status of heavy metals using various evaluation indicators and identify potential ecological risks, and (3) to identify the possible sources of heavy metals in the sediments of river–reservoir systems. This work is intended to provide new insights into the pollution status of heavy metals in the Feiyun River basin and similar river–reservoir systems, and to provide a scientific basis and necessary data support for water environmental protection research, thereby promoting healthy ecosystem development and decision-making by government departments.

## 2. Materials and Methods

### 2.1. Study Area and Sampling

**Figure 1:**
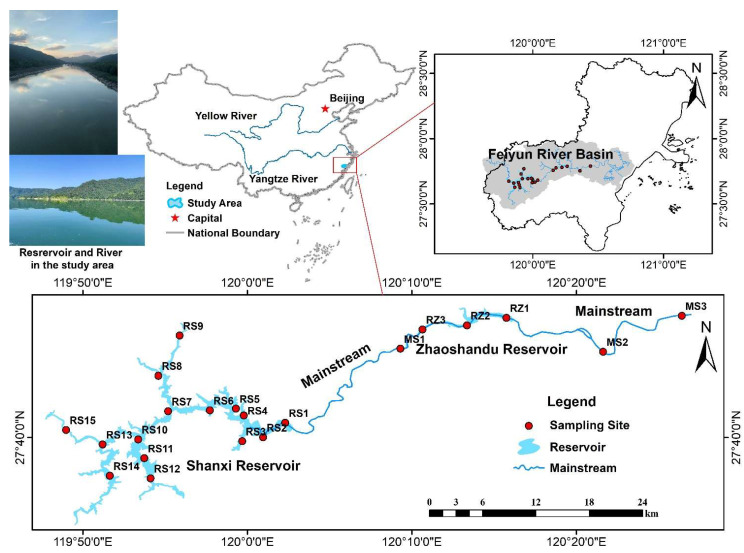
Location, rivers, and sampling sites of the Feiyun River Basin, China.

The Feiyun River Basin (119°35′~120°40′ E, 27°28′~28°00′ N) is located in the southeast of China and is the fourth-largest river in Zhejiang Province [27]. The mainstream of the Feiyun River is 191 km long and has a total basin area of 3712 km^2^. The terrain of the basin is high in the west and low in the east (Figure 1). The general trend of the mainstream is from west to east and into the East China Sea. Feiyun River is rich in water resources and has built Shanxi Reservoir (RS), Zhaoshandu Reservoir (RZ), and other key water conservancy projects, which are the water supply source for Wenzhou City’s population of 5 million. Due to historical reasons, such as man-made river basin development, the water environment in some parts of the Feiyun River has been destroyed. With the ecological destruction and soil erosion, heavy metals will flow into the river and reservoir with runoff, and the water environment of the Feiyun River Basin will face certain risks.

Field sampling was conducted in September 2021. According to the existing water area and topographic conditions of the Feiyun River Basin, and comprehensively considering road accessibility and regional representativeness, 21 sampling sites including the mainstream and main tributaries were selected, including 15 sites in Shanxi Reservoir and 3 sites in Zhaoshandu Reservoir, 3 sites on the mainstream of the Feiyun River (Figure 1). The specific method of the above sampling sites deployment refers to the “Technical Guidelines for River and Lake Health Assessment” (SL/T793-2020 (http://gjkj.mwr.gov.cn/jsjd1/tzgg_3/202006/t20200608_1407350.html (accessed on 5 September 2020)). At each site, 0~10 cm surface samples of river–reservoir system sediments were collected using a grab bucket, stored in polyethylene bags, sealed and transported to the laboratory refrigerated, and stored at −30 °C for testing.

### 2.2. Laboratory Analysis and Quality Control

The sediment samples were freeze-dried to remove impurities, and ground through a 100-mesh sieve for use, and 20 mg of the sample to be tested was weighed and placed in a closed digestion tank, and aqua regia (hydrochloric acid: nitric acid = 3:1) was added for digestion through plasma mass spectrometry (ICP-MS) determination. To ensure the accuracy of the analysis results, an average of every 10 samples was randomly selected as a parallel sample during the analysis process, and a blank experiment was performed at the same time. The relative standard deviations of the determination results of each heavy metal element were all less than 5%, which met the precision requirements stipulated by the state. Thirteen heavy metals were detected in all samples, but Cr and Se were not detected in some samples.

### 2.3. Data Statistical Analysis

#### 2.3.1. Geo-Accumulation Index

The Geological Accumulation Index (Igeo) proposed is a quantitative index used to evaluate the pollution degree of heavy metals in aquatic environment sediments, mainly quantifying metal pollution caused by human activities, natural geological and geographical processes [28]. Igeo is calculated according to the following formula:

Igeo = log_2_(C_i_/(K × B_i_)) (1)

where: C_i_ is the measured value of the heavy metal in the examined samples, B_i_ is the background value of the soil measured at a distance, and the arithmetic mean of the background value of each element of the soil in Zhejiang Province is used in this study [29]. Chinese soil background values are also given in Section 3.1 [30]. K is the correction factor to take into account the variation of the background value caused by rock formation and human activities, and the value is 1.5. Igeo can be classified into 7 levels: Class 0 (Igeo ≤ 0, unpolluted); Class 1 (0 < Igeo < 1, lightly polluted); Class 2 (1 < Igeo < 2, moderately polluted); Class 3 (2 < Igeo < 3, moderately severely polluted); Class 4 (3 < Igeo < 4, severely polluted); Class 5 (4 < Igeo < 5, severely extremely pollution); Class 6 (Igeo ≥ 5, extremely pollution).

#### 2.3.2. Enrichment Factor

Heavy metals are easily enriched in fine-grained sediments and are affected by the “grain size effect”, and the enrichment coefficient method can effectively correct the effects of changes in sediment particle size and mineral composition on the content of heavy metals [31,32,33]. The elements generally used for heavy metal standardization are Al, Fe, Mn, Si, Ti, Zr, etc. [34]. In this study, the inert element Mn was selected as the reference element, and the enrichment coefficient method was used to distinguish the natural and man-made pollution of heavy metals and to evaluate the degree of pollution. The formula for calculating the enrichment factor EF is:

EF = (M/X)*_sample_*/(M/X)*_background_* (2)

where (M/X)*_sample_* is the ratio of heavy metal elements to reference elements in the target sediment, and (M/X)*_background_* is the ratio of heavy metal elements to reference elements in the background sample. Generally, EF < 1.5 suggests that an element is entirely controlled by natural processes, and 1.5 < EF < 3, 3 < EF < 5 and 5 < EF < 10 are interpreted as minor, moderate, severe, and very severe sediment contamination, respectively [31].

#### 2.3.3. Evaluation of Potential Ecological Risk Index

The potential ecological risk index method (RI) [35] is a commonly used method to assess the risk of heavy metals in water sediments. It comprehensively considers the toxicity of heavy metals, the sensitivity of the environment to heavy metals, and the synergy of various heavy metals and sets the toxicity response coefficient to evaluate heavy metals. It is calculated as follows:

E*_r_^i^* = T*_r_^i^* × (C_i_/B_i_)) (3)

RI = ∑E*_r_^i^* (4)

where RI is the multi-factor comprehensive potential ecological risk index; E*_r_^i^* is the single-factor hazard index; T*_r_^i^* is the toxicity response coefficient, which reflects the toxicity level of heavy metals and the sensitivity of organisms to heavy metal pollution. The potential ecological risk factor and ecological risk index were calculated using standardized heavy metal toxicity coefficients for the 12 collected heavy metals based on the relevant research results of other scholars and the actual situation of the collected samples in this study [35]. By referring to the recommended value of toxicity coefficients for heavy metals [36], the toxicity response coefficients of Cr, Ni, Cu, Zn, As, Cd, Pb, Mn, V, Co, Mo, and Sb are 2, 5, 5, 1, 10, 30, 5, 1, 2, 5, 15, and 40, respectively. C_i_ is the measured value of the heavy metal in the samples; B_i_ is the background value of the measuring element, and the classification of the potential ecological risk coefficient of heavy metals and the ecological risk index RI are shown in Table 1 [37].

#### 2.3.4. Principal Component Analysis (PCA)

Principal Component Analysis (PCA) uses mathematical dimensionality reduction or feature extraction methods to linearly transform the original correlated variables and extracts a small number of important variables that are not correlated with each other and use fewer representative variables and factors to explain the main information of most variables and to speculate on possible sources of pollution. In this study, PCA was performed on eleven metals in the sediments in the watershed, and two principal components were extracted [38,39].

All statistical analyses were performed using the Origin2021 version, R 4.1.0 version, and SPSS 22.0 version.

## 3. Results and Discussion

### 3.1. Detection and Variation Characteristics of Heavy Metals in Surface Sediments

The concentration range and distribution characteristics of 15 heavy metals, including Cr, Ni, Cu, Zn, As, Cd, Pb, Mn, V, Co, Mo, Sb, W, Fe, and Se, in the surface sediments of reservoirs and rivers in the Feiyun River basin, were calculated and studied, and the statistical results are shown in Table 2. Except for Cr, Ni, Cu, V, Co, and W, the average concentration of other elements exceeded the background value, among which the average concentration of As, Sb, Mo, and Cd was 33.3, 6.89, 6.27, and 6.06 times the background value, respectively, while Zn, Mn, and Pb are 3.87, 1.35 and 1.26 times, respectively. Mo in all sampling sites exceeded the background value, and the concentrations of As, Cd, and Sb in 95.2% of the sampling sites exceeded the background value, indicating that these metal elements exceeded the standard seriously. The coefficient of variation (CV) reflects the degree of variation in the regional distribution of heavy metals in surface sediments. The larger the coefficient of variation, the higher the impact of human activities on the regional distribution of heavy metals. The variation coefficients of Cd, Zn, Se, Sb, Mn, As, and Cr are 117.67%, 156.26%, 135.85%, 119.63%, 117.67%, 111.29%, and 100.78%, respectively, which belong to extremely high variation, The spatial distribution of these species varied widely, and some sample sites were more seriously contaminated, possibly with point source input; the variation coefficients of Ni, Pb and Mo are greater than 0.5, and the coefficients of variation for other metals ranged from 0.21 to 0.41, which belonged to moderate variation, which indicates the presence of varying degrees of surface source pollution.

### 3.2. Spatial Distribution Characteristics of Heavy Metals in Surface Sediments

**Figure 2:**
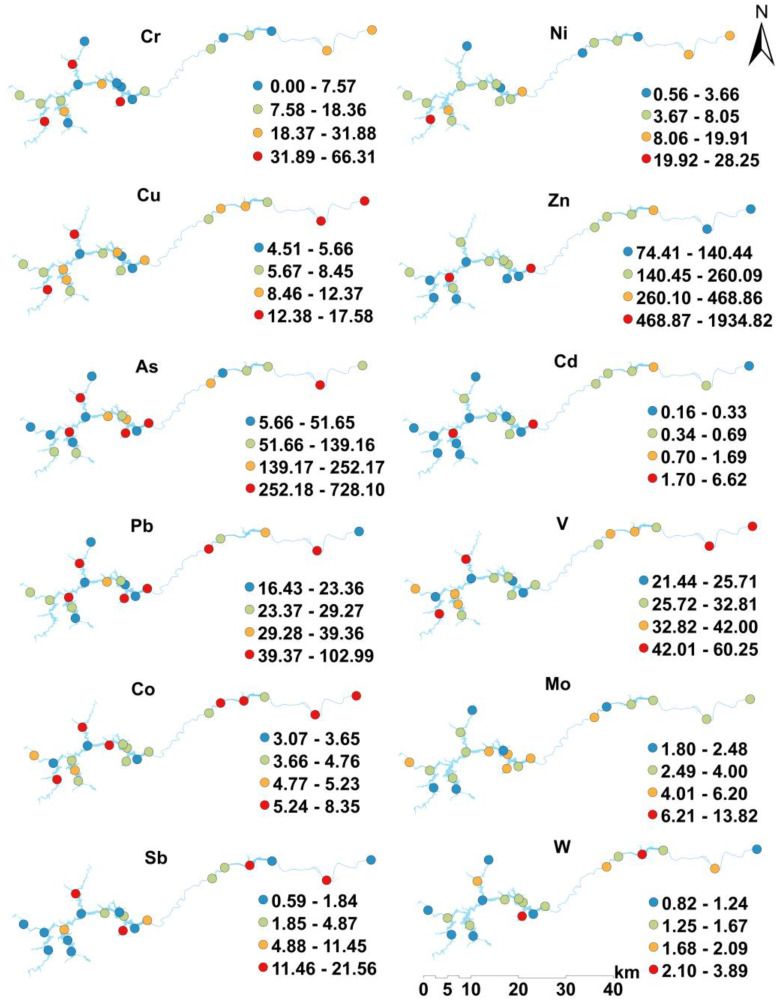
Spatial distribution characteristics of heavy metal concentrations in sediments of Feiyun River Basin.

The spatial distribution of heavy metal concentrations in surface sediments in the Feiyun River Basin is shown in Figure 2. Among them, the spatial distribution of Cu, V, and Co, is relatively consistent, and the high concentrations areas are the mainstream of Feiyun River and the northwest of Shanxi Reservoir; the high values of Ni and Sb concentrations appear in the northern part of the basin; the highest values of Zn, Cd, Mo, Pb, and W appear in the north-central and central area of Shanxi Reservoir; the highest values of Cr and As appear near the dam site of Shanxi Reservoir. Sampling site 8 of the Zhaoshandu Reservoir, the sampling site 18 of the Zhaoshandu Reservoir, and the sampling site 20 of the Feiyun River mainstream (RM) have the highest heavy metal concentrations in the three regions respectively, and sampling site 9 in the northern Feiyun River Basin has relatively low heavy metal concentrations.

### 3.3. Characteristics and Ecological Risk Assessment of Heavy Metal Pollution in Surface Sediments

#### 3.3.1. Evaluation by the Geo-Accumulation Index (Igeo)

**Figure 3:**
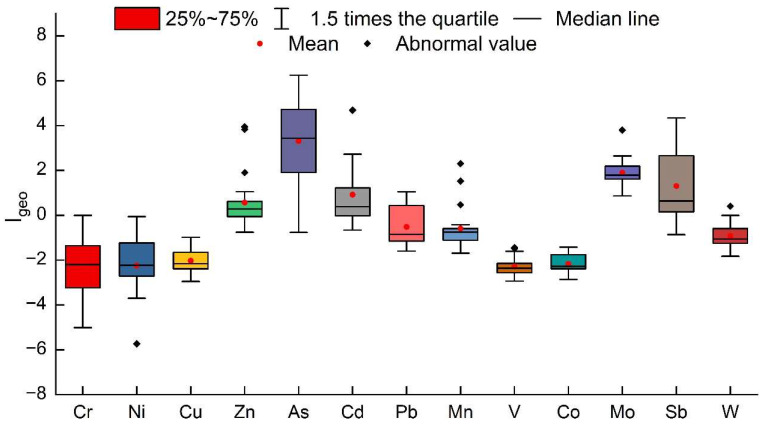
Boxplot of heavy metal geological accumulation index (Igeo) in surface sediments of the Feiyun River Basin.

The Igeo statistical results are shown in Figure 3 and Table 3. The order of the pollution levels of heavy metals in the surface sediments of the Feiyun River Basin is: As > Sb > Mo > Cd > Zn > Mn > Pb > W > Ni > Cu > Co > V > Cr. Among them, As has the highest pollution level, with an average Igeo value of 4.47. Overall, the average pollution level is severely polluted (Class 5). The As pollution in the west of Shanxi Reservoir is relatively light. The average Igeo value of Sb is 2.20, and the pollution level is moderately severely polluted (Class 3). Among them, the Shanxi Reservoir is less polluted in the central and western regions, and the mainstream of the Feiyun River is severely polluted by Sb. The average Igeo values of Mo, Cd, and Zn are 2.06, 2.01, and 1.37, and the pollution levels of the whole basin are Class 3 and Class 2, respectively. The average Igeo values of other heavy metal elements are less than 0, and the pollution conditions belong to Class 0, with no pollution.

The river–reservoir system of the Feiyun River Basin is divided into Shanxi Reservoir, Zhaoshandu Reservoir, and the mainstream of the Feiyun River. From the perspective of various regions, the highest value of Zn and Cd is in Zhaoshandu Reservoir, which is Class 2; the highest value of As is in the mainstream area of Feiyun River, which is Class 5; the highest value of Sb is Class 3, which appears in Shanxi Reservoir.

#### 3.3.2. Evaluation by Potential Ecological Risk Index (RI)

**Figure 4:**
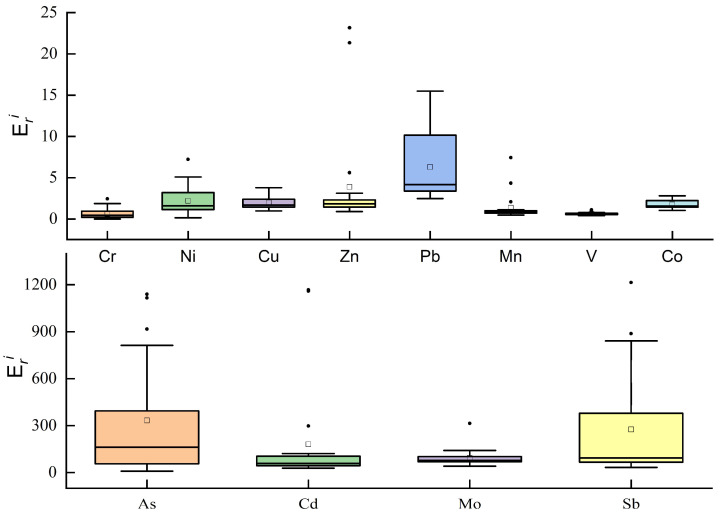
Boxplots of individual potential ecological risks of heavy metals in surface sediments.

**Figure 5:**
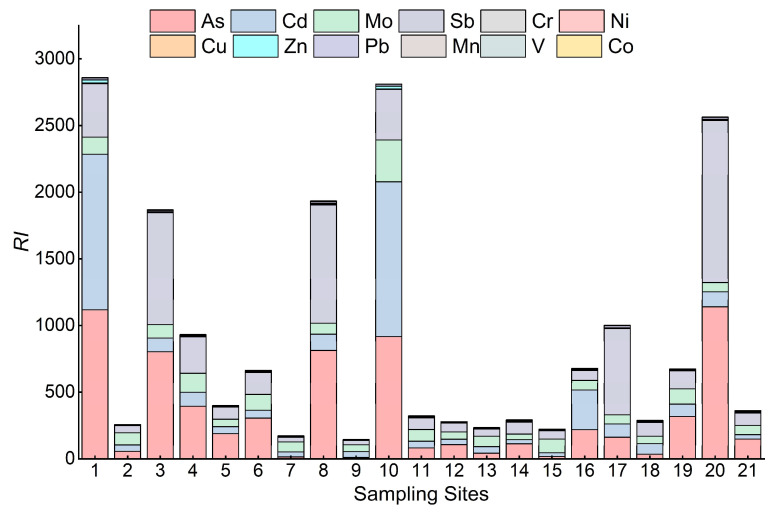
Comprehensive ecological risk index of each sampling sites.

In a previous study on the evaluation of heavy metal pollution in the evaluation with the potential ecological hazard index method, the toxicity response coefficients of a total of 12 metal elements, Cr, Ni, Cu, Zn, Pb, Mn, V, Co, As, Cd, Mo, and Sb, were derived in conjunction with the heavy metal contents detected in this study. The results of RI values of heavy metals calculated according to Equations (3) and (4) are shown in Figure 4 and Figure 5, and Table 3. The order of the mean values of 12 various heavy metals in the surface sediment species of the Feiyun River basin was As > Sb > Cd > Mo > Pb > Zn > Ni > Cu > Co > Mn > V>Cr, and the mean value of As was 332.8, with a single potential ecological risk index of very high. The values of Sb range from 33.0 to 1214.7, and the average value is 275.5, the potential ecological risk is high; the potential ecological risks of Cd and Mo are high risk and considerable risk respectively; the potential ecological risks of other metal elements are low risk.

From the perspective of the whole watershed, As and Cd have the highest potential risks in the basin. Among them, the As and Cd contents of the mainstream of Feiyun River and Shanxi Reservoir have the highest potential risks. The highest, Sb element showed medium risk in Zhaoshandu Reservoir, and was considered a considerable risk in other areas; the potential ecological risk of the remaining heavy metals was low.

The range of RI values is 144.5~2859.7, and the mean value is 902.7. The integrated ecological risk level of sampling site 1 at the dam site of Shanxi Reservoir is the highest, followed by sampling site 10. The integrated ecological risk index of sampling sites 20 and 17 at the mainstream of Feiyun River and Zhaoshandu Reservoir were the highest in the region with 2564.5 and 1001.7, respectively, indicating that heavy metal contamination of sediments in some parts of the Feiyun River basin poses a potential risk to the ecosystem and relevant measures must be taken to mitigate heavy metal pollution. In terms of the average contribution of each heavy metal to the integrated potential ecological risk, As, Sb and Cd were the main ecological risk contributors, with contributions of 36.9%, 30.5%, and 20.1% to the integrated potential ecological risk index, respectively. It can be seen that the As content of surface sediments in the Feiyun River basin is high, and although the Sb and Cd contents are low, the potential ecological risk level is high, indicating the existence of a certain degree of contamination, which should attract the attention of relevant departments.

#### 3.3.3. Evaluation by Enrichment Factor (EF)

**Figure 6:**
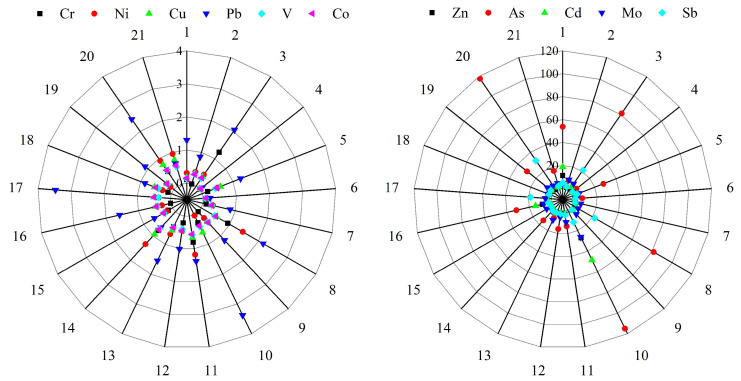
Polar plot of heavy metal enrichment factor (EF) values in the surface sediments of the Feiyun River Basin.

The average EF values of the heavy metals detected in this study ranged from 0.33 to 31.15 (Figure 6), where Pb (1.36) > Ni (0.50) > Cu (0.45) > Co (0.40) > V (0.37) > Cr (0.33), and the average EF values did not exceed 1.5, indicating that the surface sediments of the Feiyun River Basin were generally not contaminated by these heavy metals. As in RS3, RS8, RS10, and MS3, the average EF values exceeded 80, and the average EFs in the three regions of the Feiyun River basin were MS (7.36) > RS (5.05) > RZ (4.22), mainly due to the high EF values of As (31.15), Mo (6.73), and Sb (6.86). EF values are high, which indicates the more anthropogenic influence of these metal elements.

### 3.4. Correlation and Source Analysis of Heavy Metals in Surface Sediments

In this section, a total of 12 heavy metal elements, Cr, Ni, Cu, Zn, As, Cd, Pb, Mn, V, Co, Mo, Sb, and W in sediments were selected for correlation analysis and source analysis by combining the main heavy metal pollution levels, toxicity coefficients and extensive factors of heavy metal research in the Feiyun River basin.

**Figure 7:**
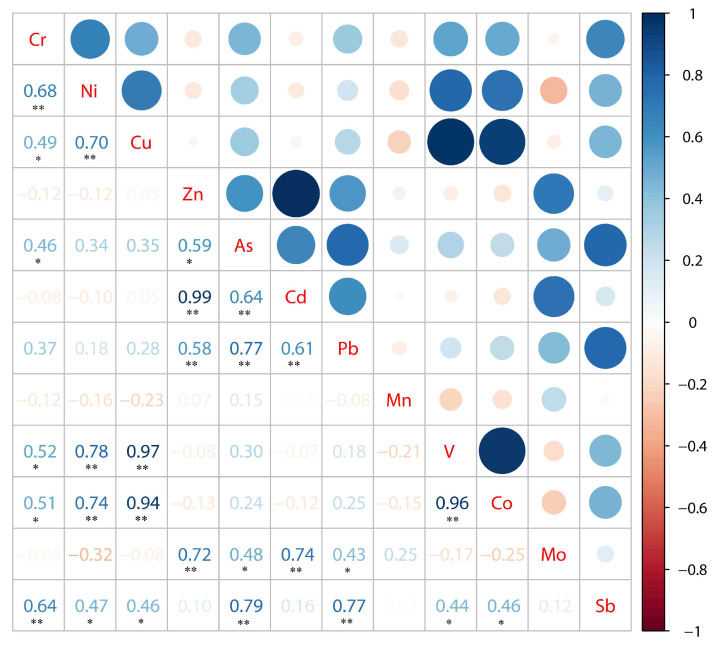
Pearson’s correlation coefficient matrix for heavy metal concentrations (** indicates that the correlation is extremely significant at the 0.01 significance level (two-tailed); * indicates that the correlation is significant at the 0.05 significance level (two-tailed).

If the correlation results between heavy metals pass the significance level test, it indicates that they may have the same source [40]. The correlation coefficient matrix between heavy metals is shown in Figure 7. There was a significant correlation between Cr and Ni and Sb (*p* < 0.01), a significant positive correlation between Ni and Cu, V and Co, a very significant correlation between Cu and V and Co, and a significant correlation between Zn and Cd, and V and Co. There is also a very significant correlation, indicating that these elements have the same origin. There are significant correlations between Sb and seven heavy metal elements, and Pb and As have similar characteristics, which indicates that the heavy metals in the Feiyun River Basin can produce complex pollution, and there are potential similar sources and migration paths.

**Figure 8:**
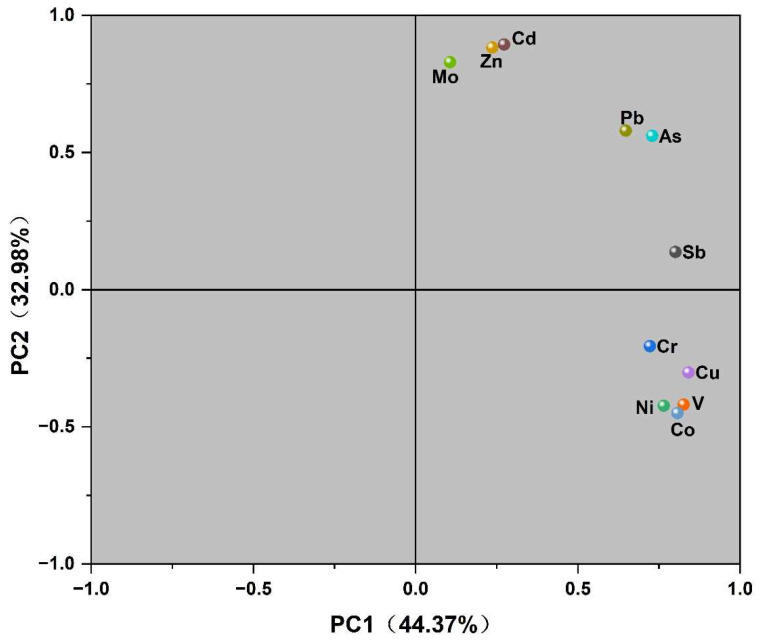
Principal component analysis loading plot of heavy metals.

To further understand the pollution sources of heavy metals in sediments of the Feiyun River Basin, principal component analysis (PCA) was used for source analysis. The Kaiser–Meyer–Olkin (KMO) test and Bartlett’s sphericity test were performed on the 12 heavy metal elements to determine the applicability of the data to PCA [41,42,43]. It is generally considered that factor analysis is suitable when KMO > 0.6. The KMO value of the 12 metal elements was 0.489, indicating that the results were extremely unsuitable. Combined with the correlation coefficient matrix between each element in Figure 7, 11 heavy metal elements except Mn were selected and tested again. The observed value of KMO value (0.624 > 0.6) and Bartletts sphericity test statistic was 326.768, the degree of freedom is 55, and the significance probability of the test is 0.000, which is less than the significance level of 0.05. The results show that the principal component analysis is effective. The specific results are shown in Figure 8 and Table 4. The eigenvalues of principal component 1 and principal component 2 are 4.880 and 3.628, respectively, the corresponding variance contribution rates are 44.368% and 32.981%, and the cumulative variance contribution rate is 77.349%, which can reflect most of the information of all data.

Among the major components1, Cu, V, Co, Sb, Ni, As and Cr all have high loadings, and the contents of Cu and Co elements are far below the background values in Zhejiang Province, with small coefficients of variation and low ecological risk, so it can be assumed that Cu and Co are mainly influenced by the natural environmental background, which is similar to the natural input law. Studies have shown that the sources of V, Sb, Ni, As and Cr are mainly industrial activities such as mining smelting, and metal processing. Wenzhou vanadium mine in the Feiyun River basin is the largest vanadium mining area in China, coupled with the developed manufacturing industry in the basin, the wastewater and waste residue generated after electroplating and metal processing is discharged into the Feiyun River. There are also studies showing that As come from wastewater irrigation, fertilizer and pesticide application, etc. The farmland in the Feiyun River basin covers an area of about 581 km^2^, and heavy metals from farmland and surrounding soil will enter the Feiyun River with rainfall-runoff, so As may also come from agricultural production.

In principal component 2, only Cd, Zn, and Mo have higher loadings (>0.8) this component, and there is a significantly strong correlation between the three elements, indicating that they have similar distribution laws [6]. The average content of Zn and Mo all exceeded the soil background value in Zhejiang Province, and the coefficient of variation was large. It can be considered that these elements are related to the introduction of industrial activities such as ore smelting in the basin.

In summary, the study concluded that heavy metals in sediments in the Feiyun River Basin were mainly affected by human activities such as sewage from domestic and agricultural activities, mining and smelting industries, and some elements were affected by natural sources.

### 3.5. Comparison of Heavy Metal Concentrations in Sediments from Other Rivers

Comparing the content of heavy metals in the surface sediments of the Feiyun River with the heavy metal content in the Oujiang River Basin in Wenzhou City, it is obvious that the average content of Zn and As is much higher than that of the Oujiang River, which may be related to the difference between the geological background and the Feiyun River [44]. It is related to human factors; compared with the heavy metals in the sediments of the Qiantang River and Dongtiaoxi River except for Zn, As and Mn, the content of heavy metals in other elements is at a lower level, which may be related to the continuous migration and accumulation of heavy metals [45,46]. Compared with the heavy metal content of other rivers and lakes in China (Table 5), it can be concluded that Zn, As, Pb, and Mn in the sediments of the Feiyun River Basin are significantly higher than those of other rivers, while the contents of Cr, Ni, and Cu are at lower levels [47,48,49,50,51].

### 3.6. Relationships between Heavy Metal Pollution and Internal Nutrients Release

The changes in heavy metal content in river surface sediments are not only controlled by natural and anthropogenic sources but also closely related to the physical and chemical properties of the sediments themselves [52,53], Several recent studies have found that heavy metal pollution may be influenced by eutrophication, with significant correlations found between heavy metal concentrations and total phosphorus concentrations in sediments in some lakes in China, implying an association in transport and similarity in their sources [6,54]. With the rapid development of urbanization and agriculture in the watershed, the watershed is subject to the hazards of eutrophication of water bodies and heavy metal pollution, and these factors intertwine to influence the deterioration of aquatic ecosystems such as rivers and reservoirs. The organic matter (OM), total nitrogen (TN), total phosphorus (TP), temperature (Temp), and oxidation-reduction potential (ORP) measured in the surface sediments of 21 sampling sites in the Feiyun River Basin were compared with the sediments at the corresponding points [55,56,57]. Pearson correlation was used for heavy metal content. According to the results in Table 6, OM only showed a significant negative correlation with Cu (*p* < 0.01), indicating that OM may not be the key factor affecting the surface heavy metal content in the Feiyun River Basin; TN has a very significant negative correlation with Cu, V, and Co, and TP has a significant negative and positive correlation with V and Co, respectively. These highly toxic heavy metal elements have parentage with TOC, indicating that sediments are affected by human activities. The impact is more obvious. In addition, the combination of organic matter and TN with more toxic heavy metals will produce more toxic complexes, which further aggravates the potential ecological harm of sediments in the Feiyun River Basin. However, there is no correlation between ORP and these heavy metal elements, indicating that the effect of OPR is limited. Total nitrogen and total phosphorus in surface sediments were indeed associated with more heavy metals, suggesting that the eutrophication of water bodies in the Feiyun River basin might be related to the concentration of heavy metals in surface sediments.

## 4. Conclusions

Concentration level and spatial distribution of heavy metals (ice, Cr, Ni, Cu, Zn, As, Cd, Pb, Mn, V, Co, Mo, Sb, W, Fe, and Se) in the surface sediments of the river-reservoir system in the Feiyun River Basin, source identification and potential ecological risks were investigated and analyzed in detail. As a result, except for Cr, Ni, Cu, V, Co, and W in the surface sediments of the river reservoir system in the Feiyun River Basin, the contents of other heavy metals all exceeded the soil background values of Zhejiang Province; the mainstream of Feiyun River > Zhaoshandu Reservoir > Shanxi Reservoir; the spatial distribution characteristics of Cu, V, Co, and Fe are similar, and other heavy metal elements have higher pollution levels in different regions. The geo-accumulation index method evaluation shows that As and Sb in the river reservoir system of Feiyun River Basin are severely polluted, and moderately severely polluted, respectively, and other heavy metals are not polluted except for Mo, Cd, and Zn; The enrichment index method shows that As and Sb are, severally, very severe sediment contamination; the potential ecological index evaluates the surface sediments in the Feiyun River Basin as a very high-risk level, the main environmental The risk factors are As, Sb, Cd, and Mo respectively; by using correlation analysis and principal component analysis, it shows that the heavy metals in surface sediments in the river-reservoir system of the Feiyun River Basin may be mainly affected by human activities such as sewage from domestic and agricultural activities, mining and smelting, and some elements are affected by natural factors. Our study provides basic information on heavy metal contamination in the basin surface sediments of an important, typical reservoir–river system and suggests that anthropogenic processes dominate heavy metal contamination of surface sediments throughout the river. As an inlet river, intensive anthropogenic activities in and around the Feiyun River basin have led to heavy metal pollution in the aquatic system. The surface sediments of rivers may carry comprehensive information about the changes in the water environment in the basin, therefore, we still need to make long-term observations of heavy metals and other physicochemical property indicators in the surface sediments of coastal rivers in southeast China, including the Feiyun River basin, as a way to study the integrated effects of climate change and human activities on environmental changes in the southeast coastal region of China.

## Figures and Tables

**Table 1:** Classification of potential ecological risk coefficient (E*_r_^i^*) and ecological risk index (RI) of heavy metals.

Potential Ecological Risk Coefficient (E*_r_^i^*)	Ecological Risk Index (RI)	Potential Ecological Risk Level Classification
<40	<150	Low risk
40~80	150~300	Medium risk
80~160	300~600	Considerable risk
160~320	>600	High risk
>320	—	Very high risk

**Table 2:** Descriptive statistics on the content of heavy metal in surface sediments (unit: mg/kg).

Heavy Metal	Min	Median	Max	Mean	SD	CV (%)	ZB ^1^	CB ^2^
Cr	0.00	11.90	66.31	16.62	16.75	100.78	54.34	65.65
Ni	0.56	6.25	28.25	8.61	6.83	79.33	19.59	29.88
Cu	4.51	7.78	17.58	9.19	3.68	40.05	23.21	28.27
Zn	74.41	152.55	1934.82	323.53	505.55	156.26	83.52	109.57
As	5.66	103.45	728.11	212.64	236.64	111.29	6.39	7.19
Cd	0.16	0.33	6.62	1.03	1.83	177.67	0.17	0.23
Pb	16.43	27.59	102.99	41.88	27.50	65.66	33.24	44.37
Mn	353.03	676.67	5637.75	1026.24	1197.22	116.66	759.00	767.74
V	21.44	31.79	60.25	35.19	11.32	32.17	109.00	71.39
Co	3.07	4.60	8.35	5.20	1.65	31.73	14.86	11.57
Mo	1.80	3.42	13.82	4.14	2.45	59.18	0.66	0.99
Sb	0.59	1.66	21.56	4.89	5.85	119.63	0.71	0.76
W	0.82	1.41	3.89	1.64	0.68	41.46	1.95	2.72
Fe	11,817.94	16,601.61	23,407.23	16,660.26	3503.46	21.03	— ^3^	—
Se	0.00	0.00	2.37	0.53	0.72	135.85	—	—

^1^ ZB is the soil background value in Zhejiang Province. ^2^ CB is the soil background value in China. ^3^ “—” means no data.

**Table 3:** Geological accumulation index method and potential ecological risk assessment results.

Heavy Metal		Cr	Ni	Cu	Zn	As	Cd	Pb	V	Co	Mo	Sb
Igeo	Mean	−2.2	−2.2	−2.0	0.6	3.3	0.9	−0.5	−0.6	−2.3	−2.2	1.9
	RS	−2.3	−2.3	−2.2	0.6	3.1	0.8	−0.6	−0.5	−2.4	−2.3	2.0
	RZ	−2.0	−2.6	−1.9	1.1	2.8	1.6	−0.1	−0.9	−2.2	−1.9	1.5
	MS	−2.1	−1.7	−1.5	−0.1	4.6	0.7	−0.4	−0.6	−1.9	−1.8	1.9
Pollution risk	WB	—	—	—	Class 1	Class 4	Class 1	—	—	—	—	Class 2
	RS	—	—	—	Class 1	Class 4	Class 1	—	—	—	—	Class 3
	RZ	—	—	—	Class 2	Class 3	Class 2	—	—	—	—	Class 2
	MS	—	—	—	—	Class 5	Class 1	—	—	—	—	Class 2
E*_r_^i^*	Mean	0.6	2.2	2.0	3.9	332.8	181.7	6.3	1.4	0.6	1.7	94.0
	RS	0.6	2.2	1.8	4.4	331.4	206.6	5.8	1.5	0.6	1.6	101.5
	RZ	0.3	1.3	2.1	3.6	138.0	158.6	8.3	0.8	0.7	2.0	65.7
	MS	0.8	3.1	2.9	1.5	534.3	80.1	6.9	1.0	0.9	2.3	84.7
Potential ecological risk	WB	—	—	—	—	V	H	—	—	—	—	C
	RS	—	—	—	—	V	H	—	—	—	—	C
	RZ	—	—	—	—	C	C	—	—	—	—	M
	MS	—	—	—	—	V	C	—	—	—	—	C

**Table 4:** Analysis of the main components of heavy metals in sediments.

Element	PC1	PC2
Cu	0.841	−0.302
V	0.826	−0.419
Co	0.807	−0.450
Sb	0.801	0.138
Ni	0.765	−0.423
As	0.729	0.561
Cr	0.722	−0.206
Pb	0.648	0.580
Cd	0.273	0.894
Zn	0.236	0.883
Mo	0.106	0.829
Eigenvalue	4.880	3.628
% of Total Variance	44.368	32.981
Cumulative %	44.368	77.349

**Table 5:** The concentration of heavy metals in sediments of different rivers in China (mg/kg).

River	Year	Cr	Ni	Cu	Zn	As	Cd	Pb	Mn	V	Co	Mo	Sb	W
FRB (this study)	2021	16.62	8.61	9.19	323.53	212.64	1.03	41.88	1026.24	35.19	5.20	4.14	4.89	1.64
Oujiang River	2014	177.9	63.2	64.5	194.3	11	—	53.3	—	—	51.1	—	—	—
Qiantangjiang River	2017	73.16	—	103.73	223.76	11.91	2.50	46.58	736.4	—	—	—	—	—
Dongtiaoxi River	2016	67.88	39.18	47.87	200.62	48.49	1.08	47.13	951.20	—	24.1	—	—	—
Taihu Lake	2015	82.3	43.9	32.8	109	—	0.55	35.1	886	—	15.8	0.62	2.37	—
Ganjiang River	2019	4.7	—	84.3	362	31.5	2.3	52.8	980.8	—	—	—	—	25.7
Dongting Lake	2018	93.47	34.47	37.98	147.19	21.23	1.91	36.05	—	—	—	—	—	—
Yangtze River	2020	83.99	—	36.45	124.21	11.20	0.77	36.40	—	—	—	—	—	—
Yellow River	2021	73.36	31.13	24.96	87.17	11.78	0.58	26.92	—	—	—	—	—	—

**Table 6:** Spearman’s correlation coefficients between metals in surface sediments and nutrients.

Nutrients	Cr	Ni	Cu	Zn	As	Cd	Pb	Mn	V	Co	Mo	Sb
OM	−0.18	−0.22	−0.44 *	−0.05	−0.08	−0.04	−0.12	0.08	−0.40	−0.38	−0.06	−0.09
TN	−0.27	−0.34	−0.68 **	0.03	−0.13	0.03	−0.12	0.20	−0.62 **	−0.59 **	0.11	−0.18
TP	0.33	0.27	0.36	−0.27	−0.09	−0.25	0.05	−0.41	0.44 *	−0.45 *	−0.17	0.22
Temp	−0.10	0.13	0.32	−0.31	−0.26	−0.32	−0.20	−0.31	0.38	0.40	−0.45 *	−0.10
ORP	0.05	0.15	0.28	−0.22	0.04	−0.18	−0.11	−0.23	0.27	0.20	−0.15	0.09

** indicates that the correlation is extremely significant at the 0.01 significance level (two-tailed); * indicates that the correlation is significant at the 0.05 significance level (two-tailed).

## Data Availability

The data that support the findings of this study are available from the corresponding author upon reasonable request.
